# Integrated Anterior, Central, and Posterior Skull Base Unit – A New Perspective

**DOI:** 10.3389/fsurg.2015.00032

**Published:** 2015-07-21

**Authors:** Yves Brand, Vicknes Waran, Abu Bakar Zulkiflee, Elizabeth Lim, Narayanan Prepageran

**Affiliations:** ^1^Department of Otorhinolaryngology, University Malaya Medical Centre, Kuala Lumpur, Malaysia; ^2^Department of Neurosurgery, University of Malaya Medical Centre, Kuala Lumpur, Malaysia

**Keywords:** anterior skull base, central skull base, extended endoscopic endonasal approaches, lateral skull base, neurotology, skull base surgery

## Abstract

The skull base is one of the most complex anatomical regions and forms the floor of the cranial cavity. Skull base surgery involves open, microscopic, and endoscopic approaches to the anterior, middle, or posterior cranial fossa. A multispecialty team approach is essential in treating patients with skull base lesions. Traditionally, rhinologists are involved in providing access to anterior skull base lesions while otologists are involved in the treatment of lesions of the posterior skull base. This is the case in most skull base centers today. In this article, we share a new perspective of an integrated skull base unit where a team of otolaryngologists and neurosurgeons treat anterior, middle, and posterior skull base pathologies. The rationale for this approach is that most technical skills required in skull base surgery are interchangeable and apply whether an endoscopic or microscopic approach is used. We show how the different skills apply to the different approaches and share our experience with an integrated skull base unit.

## Introduction

The skull base is one of the most complex anatomical regions within the human body with a variety of vital structures all within close proximity. It separates the brain from the facial skeleton and forms the floor of the cranial cavity. Skull base surgery requires a multidisciplinary team approach. The two members of this team include the neurosurgeon and the ENT surgeon. Their mutual support of each other is essential for success (1). Traditionally from an otolaryngologist’s point of view, rhinologists perform anterior skull base surgery while otologists perform the approaches to the lateral skull base.

Rhinologists traditionally use an endoscope and have profound knowledge of the anatomy of the paranasal sinuses. However, endoscopic skull base surgery and extended endoscopic endonasal approaches are a relatively young surgical field. The first purely endoscopic approach to the sella to remove a pituitary tumor was published in 1992 by Janowski et al. (2). As the field progressed, transnasal endoscopic skull base surgery emerged, and extended endoscopic endonasal approaches were developed from the sella to the anterior and posterior cranial fossa [reviewed in Ref. (3)]. By the turn of the century, Thaler and colleagues described the utility of the endoscope for anterior skull base surgery (4). They reported an endoscopic-assisted removal of a sinonasal tumor with intracranial extension. The direct visualization with the endoscopic technique facilitated a safer and more accurate removal of the tumor without sacrificing vital neurovascular structures. In 2001, Casiano described the first purely endoscopic endonasal anterior skull base resection by illustrating five purely endoscopically treated esthesioneuroblastomas (5).

Conversely, otologists are trained in using the microscope and have experience in the anatomy of the temporal bone. William House applied microsurgical techniques to remove acoustic tumors in the early 1960s (6). Today, translabyrinthine, retrosigmoidal, and middle fossa approaches are used to address these tumors and they have been proven to be safe with minimum mortality and morbidity. Fisch, Glasscock, and Jackson were among the pioneers who advanced the field in the 1970s and 1980s in developing approaches to treat glomus tumors and other pathologies surgically (7, 8).

The difference in the developments of surgeries, from sinus surgery to anterior skull base surgery and from middle ear surgery to lateral skull base surgery became separate fields. As a result, most of the skull base centers have different teams treating different skull base lesions. Otologists perform microsurgery of the lateral skull base, and rhinologists perform transnasal endoscopic skull base surgery. There are significant differences between surgeries with the microscope in the temporal bone compared to endoscopic endonasal approaches. However, many of the skills required to perform skull base surgery are interchangeable and apply to both endoscopic endonasal and also microscopic lateral skull base surgery.

The aim of this article is to present a new perspective to skull base surgery from an otolaryngologist’s point of view. We report on our experience of an integrated skull base unit where the same team is treating skull base lesions, whether this involves an approach to the posterior skull base using a microscope or an extended endoscopic endonasal approach to the anterior or central skull base. We highlight the fact that most of the skills needed to perform skull base surgery are interchangeable. Therefore, we believe that an integrated skull base unit is an alternative to the current setup in most skull base centers today.

## Interchangeable Skills

Several skills are required to perform skull base surgery. A profound knowledge of anatomy is fundamental. Only a detailed knowledge of anatomy enables the surgeon to analyze pre-operative imaging studies in order to decide on what surgical approach is most suitable. During surgery, detailed anatomical knowledge is required to avoid complications. However, in addition to a detailed knowledge of anatomy, several technical skills are vital to perform skull base surgery. We believe that most of the skills required are interchangeable and apply both to endoscopic endonasal and microscopic lateral skull base surgery.

The surgery itself is always a collaborative effort between the neurosurgeon and ENT surgeon. In our institution, most of the drilling is performed by the ENT surgeon with the neurosurgeon assisting and guiding, while the intradural work is performed by the neurosurgeon assisted by the ENT surgeon who is holding the endoscope. Once the dura is reached, the neurosurgeon can concentrate on the intradural work without experiencing micro-tremors in his hand since the ENT surgeon performed the drilling. This collaborative approach also holds true for lateral skull base surgery when using a microscope. ENT surgeons are usually more familiar with the surgical anatomy of the sinuses in extended endoscopic endonasal approaches to the skull base or bony anatomy of the temporal bone. The skills acquired in using a drill in otology are also interchangeable and apply to both endoscopic and microscopic skull base surgery. When handling a drill, familiarity with the tactile feedback and the ability to appreciate the changes of sound when the dura is reached are skills that allow efficient and fast drilling. In fact, this is not only the case for drilling, but also for a variety of instruments used in both endoscopic and microscopic skull base surgery.

Management of intraoperative hemorrhage during skull base surgery is essential. The tools used to control bleeding include diathermy/electrocautery, vessel clips, hemostatic agents (e.g., surgical, gelfoam, flowseal, etc.), bone wax, and hot saline irrigation. The principles of controlling intraoperative hemorrhage apply whether or not an endoscope or a microscope is used.

One can argue that rhinologists are more familiar with the use of an endoscope. This certainly holds true when compared to a surgeon who is only involved in microscopic ear surgery. Endoscopic sinus surgery was introduced in the 1970s by Messerklinger and further advanced in the following years by Stammberger in Europe (9). In 1985, David Kennedy and colleagues introduced endoscopic sinus surgery to the United States (10). However, it should be noted that many rhinologist still used the microscope to perform endoscopic sinus surgery in 1992, when the first purely endoscopic approach to the sella was performed (11). Interestingly, as sinus surgery has transferred from the microscope to the endoscope, there are trends that involve certain middle ear surgeries that can be performed using an endoscope as well (12). Today, the younger generation of surgeons is trained using either an endoscope in sinus surgery or a microscope for ear surgery.

Training of the future leaders in our field is essential for progress and new innovations. As a result, many institutions offer fellowship programs in different subspecialties. However, fellowship training programs in otolaryngology either offer subspecialty training in otology/lateral skull base surgery or in rhinology/endoscopic anterior skull base surgery. As a result, different teams usually treat skull base pathologies. However, skull base pathologies are rare conditions, and in many centers the workload allows an alternative approach where the same team performs the surgery regardless to the approach used. We believe that this approach allows higher exposure to skull base pathologies. However, the idea of an integrated skull base unit needs fundamental changes in training of future skull base surgeons and adaptations of current fellowship programs. Moreover, fewer surgeons should be trained in skull base surgery, thus limiting the training to those surgeons who are most skilled and have a strong interest in academic medicine.

## Our Experience with an Integrated Skull Base Unit

Our integrated skull base unit started in 2007. The main ENT surgeon and neurosurgeon had already been closely working together in cases involving the lateral skull base – mostly translabyrinth and retrocochlear approaches to the cerebellopontine angle prior to its start. As extended endoscopic endonasal approaches became the new frontier in skull base surgery, they started an integrated approach also involving endoscopic skull base surgery. The team first started with pituitary surgeries, and gradually worked with more complex surgeries. Today, the team is performing complex cases (Level IV and Level V according to Kassam) (13). Training is essential in skull base surgery; the main neurosurgeon and ENT surgeon are both fellowship trained. Prior to embarking on extended endoscopic endonasal approaches, the main ENT surgeon already had extensive experience in rhinology. Moreover, both surgeons are familiar with open approaches to the skull base.

**Figure 1:**
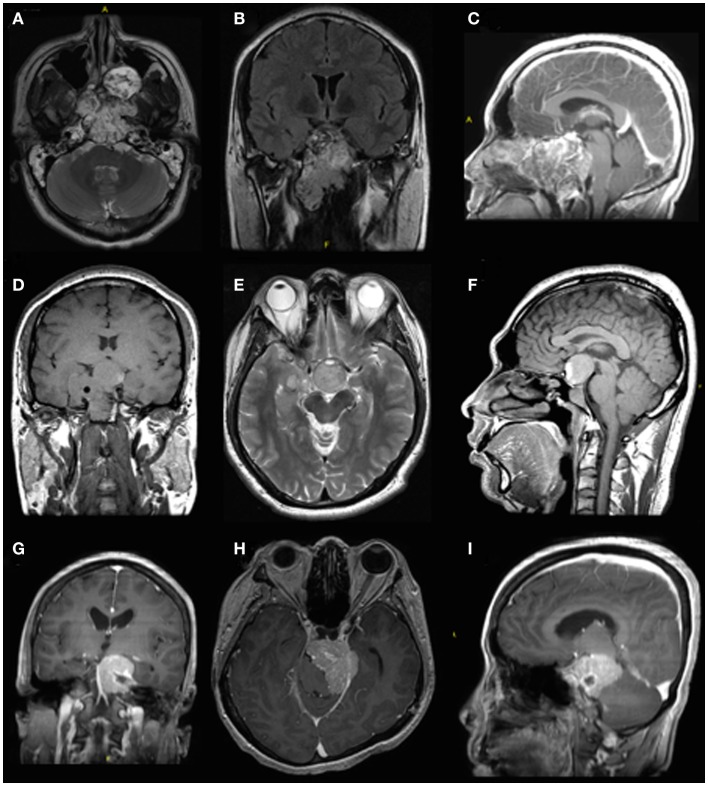
**Pre-operative imaging of different skull base pathologies**. The first case **(A–C)** is a 30-year-old male with a clival chordoma. The second case is a 36-year-old male with a recurrent pituitary macroadenoma **(D–F)**. Both these cases were treated with an extended endoscopic endonasal approach. The third case **(G–I)** is a 52-year-old male with a meningioma in the left cerebellopontine angle. A retrosigmoidal approach was used in this case.

Today, the team treats a total of approximately 150 skull base cases each year using an integrated team approach. This does not include tumors that are addressed with a craniotomy approach. The majority of cases (approximately 80%) involve extended endonasal endoscopic approaches. As in other centers, the majority of cases involve transsphenoidal resection of pituitary tumors (13). About 20% of the cases involve lateral approaches to the skull base – mostly acoustic schwannomas using a retrolabyrinthine or translabyrinthine approach. Figure 1 illustrates some of the cases treated. During the last 8 years, this integrated approach has proven to be successful in our setting. The caseload allows the same team to manage the cases while also gaining expertise in the field. We found that many of the skills involved in skull base surgery are interchangeable as described above. Most importantly, the morbidity and mortality are in the same range as in well-established centers where separate teams perform extended endoscopic endonasal approaches and lateral skull base surgery (13). Moreover, we have established a hands-on fellowship program where future skull base surgeons are exposed to all the skull base cases and where we emphasize utilization of the interchangeable skillsets.

We believe that the idea of an integrated skull base unit is a new perspective and alternative to the settings found in most centers today. We are aware that this alternate approach is not suitable for all centers and that adequate training and exposure are essential. However, we found the concept of an integrated skull base unit suitable and successful in our setting.

## Conflict of Interest Statement

The authors declare that the research was conducted in the absence of any commercial or financial relationships that could be construed as a potential conflict of interest.
